# Editorial: Mechanisms of Preservation and Change in Archaeal Genomes

**DOI:** 10.3389/fmicb.2021.722509

**Published:** 2021-07-29

**Authors:** Dennis W. Grogan

**Affiliations:** Department of Biological Sciences, University of Cincinnati, Cincinnati, OH, United States

**Keywords:** archaea, topoisomerases, homologous recombination, argonaute proteins, DNA primase, DNA polymerases, endonucleases, mismatch repair

In 1977 Carl Woese identified a “primary kingdom” of life which he termed Archaebacteria (Woese and Fox, 1977), and over the intervening decades, the distinctiveness of these organisms (now known as Archaea) has motivated some of microbiology's most interesting research. Today, as genome analyses probe microbial diversity more broadly and deeply than has been possible before, new archaeal groups continue to be discovered and the complex relationships underlying unicellular life on earth continue to be examined (Liu et al., 2021). Consistent with various molecular phylogenies, archaea have been found to share certain cellular features only with eukaryotes, other features only with bacteria, and yet others only with other archaea. The phylogenetic and physiological divergence that separates them from nearly all intensively studied model organisms further argues that archaea harbor additional examples of life's functional diversity which remain undiscovered.

The situation with respect to genome maintenance is particularly intriguing: to replicate their DNA, archaea employ proteins homologous to those of eukaryotes, whereas the cellular context and genetic organization of archaeal chromosomes are more bacterial in nature (Kelman and Kelman, 2014). The genome represents both the history and the functional potential of a microbial cell; preserving it over many generations is a basic requirement of biological success and requires co-operation among several distinct enzymatic systems. Conversely, genome alterations, which represent the first step of evolutionary adaptation and speciation, may result from simple chemical accidents, but most involve complex enzymatic processes. Analyzing the processes which shape the genomes of archaea promises not only to yield new insights into early cellular evolution and a clearer understanding of how modern archaea diversify but also improved genetic methods for archaea and widely useful technologies based on archaeal proteins.

The rewards and risks of determining experimentally how archaea promote or control genetic processes are heightened by the extreme growth conditions of many archaea and the difficulty (or often, lack) of laboratory cultivation for many of them. In the Research Topic on mechanisms of preservation and change in archaeal genomes, researchers from around the world present experimental studies investigating how archaea carry out basic genetic processes. Each of the seven contributions (one review and six original research reports) enlarges the basis for ongoing efforts to reconstruct the early evolution of cells and develop new methods of archaeal genetics and biotechnology.

Topoisomerases, for example, support diverse DNA transactions which affect genome structure and gene expression. Garnier et al. provide a clear and authoritative guide to archaeal topoisomerases which they dedicate to the memory of Maria Ciaramella and her research on archaeal DNA repair. After explaining topoisomerases in mechanistic terms, the review outlines the molecular diversity and biology of the archaeal enzymes, including their significance for challenges specific to cells growing under extreme conditions (Garnier et al.).

One of the processes that operates in all cells to determine genome structure is homologous recombination, which supports genome preservation through repair of double-strand breaks and accurate bypass of unrepaired damage, and genome alteration through strand or sequence transfer between non-equivalent regions of shared sequence. Archaea encode characteristically “archaeal” homologs of bacterial or eukaryotic recombination proteins, but the functional properties of recombination have been studied in relatively few archaea. Wasser et al. used the technique of protoplast fusion to produce artificially heterozygous *Haloferax volcanii* cells, and recovered recombinants without prior genetic selection. Their results indicate that non-reciprocal recombination (i.e., gene conversion) is very efficient in this species and forms tracts that typically cover multiple genes.

The value of directing natural defense systems to disrupt expression of specific genes or the genes themselves has been well-demonstrated in eukaryotic organisms by RNA interference (RNAi) and later by use of prokaryotic CRISPR-Cas proteins. Argonaute (Ago) proteins play a central role in RNAi, but homologous proteins occur in diverse bacteria and archaea. Guo et al. analyzed functional properties of the Ago homolog encoded by the hyperthermophilic archaeon *Ferroglobus placidus* and found that relatively short guide DNAs directed the site-specific cleavage of target DNA at temperatures up to 99 C (Guo et al.). The properties of this endonuclease shed new light on the potential roles of Ago proteins in prokaryotes and the potential uses of the archaeal enzymes in DNA or genome engineering.

Perhaps the most basic process capable of modifying genome sequences is that of DNA replication. DNA primases are central to replication and define two major types: the DnaG (bacterial) type and the AEP (archaeo-eukaryotic primase) type. Bergsch et al. analyze an AEP that supports replication of the *Sulfolobus* plasmid pRN1 in order to identify how this primase enforces its characteristic 8-nt primer length (Bergsch et al.). The results implicate molecular interactions among the nascent primer and multiple enzyme domains, but not the mechanisms that have been proposed previously based on study of other AEPs. This archaeal enzyme may therefore represent the first demonstration of a previously unknown strategy to define primer length.

DNA polymerases similarly represent distinct protein families distributed among cellular organisms, viruses and some plasmids. B-family members are the most common DNA polymerases among archaea, and usually include the replicative polymerases among the Sulfolobales and other crenarchaeotes. Two independent studies now confirm that the PolB2 and PolB3 polymerases of these archaea are not essential but nevertheless contribute to some aspects of DNA-damage repair or tolerance. Miyabayashi et al. analyzed *S. acidocaldarius* deletion strains and observed various phenotypes when both the PolB2 and PolB3-encoding genes were deleted. In another study, Bohall and Bell confirmed the dispensability of the two corresponding polymerases of *S. islandicus* and observed a similar but distinct pattern of phenotypes that included increased hydrogen peroxide survival in PolB mutants (Bohall and Bell).

Finally, one of the enduring mysteries of extremely thermophilic and hyper-thermophilic archaea regards the high accuracy of their genome replication despite the lack of MutS/MutL-homologous pairs, which are critical to canonical post-replicational mismatch repair (Grogan, 2015). Ahmad et al. investigated the *S. islandicus* and *S. acidocaldarius* homologs of known mismatch-specific endoncleases (EndoMS) and confirmed that both of the *Sulfolobus* proteins cleave duplex DNA at mismatches (Ahmad et al.). Furthermore, deletion of the *S. islandicus* gene increased the frequency of spontaneous mutation dramatically, whereas over-expressing this gene impaired culture growth, cell division, and chromosome replication.

All of these articles provide valuable perspectives on genetic processes that maintain, and sometimes modify, the genomes of archaea. The questions they address relate to the molecular strategies underlying basic genetic processes, and the results they report provide new insights into functional dimensions of the evolutionary diversity that has been revealed through analyses of archaeal genomes.

## Author Contributions

The author confirms being the sole contributor of this work and has approved it for publication.

## Conflict of Interest

The author declares that the research was conducted in the absence of any commercial or financial relationships that could be construed as a potential conflict of interest.

## Publisher's Note

All claims expressed in this article are solely those of the authors and do not necessarily represent those of their affiliated organizations, or those of the publisher, the editors and the reviewers. Any product that may be evaluated in this article, or claim that may be made by its manufacturer, is not guaranteed or endorsed by the publisher.
